# Coding practice in national and regional kidney biopsy registries

**DOI:** 10.1186/s12882-021-02365-3

**Published:** 2021-05-24

**Authors:** Amélie Dendooven, Han Peetermans, Mark Helbert, Tri Q. Nguyen, Niels Marcussen, Michio Nagata, Loreto Gesualdo, Agnieszka Perkowska-Ptasinska, Cristina Capusa, Juan M. López-Gómez, Colin Geddes, Myrurgia A. Abdul-Hamid, Mårten Segelmark, Rosnawati Yahya, Mariela Garau, Russell Villanueva, Anthony Dorman, Sean Barbour, Ronald Cornet, Helmut Hopfer, Kerstin Amann, Sabine Leh, On behalf of the Kidney Biopsy Codes for Pathologists project (www.kibico.org)

**Affiliations:** 1 Ghent University Hospital, Ghent, Belgium; 2 Antwerp University, Antwerp, Belgium; 3 ZNA Middelheim Hospital, Antwerp, Belgium; 4 University Medical Center, Utrecht, The Netherlands; 5 Odense University Hospital, Odense, Denmark; 6 University of Tsukuba Hospital, Tsukuba, Japan; 7 University of Bari Aldo Moro, Bari, Italy; 8 Medical University of Warsaw, Warsaw, Poland; 9 Carol Davila University of Medicine and Pharmacy, Bucharest, Romania; 10 Hospital General Universitario Gregorio Marañón, Madrid, Spain; 11 Glasgow Renal and Transplant Unit, Glasgow, UK; 12 Maastricht University Medical Center, Maastricht, The Netherlands; 13 Lund University, Lund, Sweden; 14 Hospital Kuala Lumpur, Kuala Lumpur, Malaysia; 15 University of the Republic, Montevideo, Uruguay; 16 National Kidney and Transplant Institute, Quezon City, The Philippines; 17 Beaumont Hospital, Dublin, Ireland; 18 University of British Columbia, Vancouver, Canada; 19 Amsterdam University Medical Center, Amsterdam, The Netherlands; 20 University Hospital of Basel, Basel, Switzerland; 21 Universitätsklinikum Erlangen, Erlangen, Germany; 22 Haukeland University Hospital, Bergen, Norway

**Keywords:** Kidney biopsy registry, Systematic review, Coding, Renal pathology, Nephropathology

## Abstract

**Background:**

Kidney biopsy registries all over the world benefit research, teaching and health policy. Comparison, aggregation and exchange of data is however greatly dependent on how registration and coding of kidney biopsy diagnoses are performed. This paper gives an overview over kidney biopsy registries, explores how these registries code kidney disease and identifies needs for improvement of coding practice.

**Methods:**

A literature search was undertaken to identify biopsy registries for medical kidney diseases. These data were supplemented with information from personal contacts and from registry websites. A questionnaire was sent to all identified registries, investigating age of registries, scope, method of coding, possible mapping to international terminologies as well as self-reported problems and suggestions for improvement.

**Results:**

Sixteen regional or national kidney biopsy registries were identified, of which 11 were older than 10 years. Most registries were located either in Europe (10/16) or in Asia (4/16). Registries most often use a proprietary coding system (12/16). Only a few of these coding systems were mapped to SNOMED CT (1), older SNOMED versions (2) or ERA-EDTA PRD (3). Lack of maintenance and updates of the coding system was the most commonly reported problem.

**Conclusions:**

There were large gaps in the global coverage of kidney biopsy registries. Limited use of international coding systems among existing registries hampers interoperability and exchange of data. The study underlines that the use of a common and uniform coding system is necessary to fully realize the potential of kidney biopsy registries.

**Supplementary Information:**

The online version contains supplementary material available at 10.1186/s12882-021-02365-3.

## Background

The percutaneous kidney biopsy is the gold standard to diagnose renal disease, especially glomerulonephritis [1]. Microscopic examination of kidney tissue gives information about diagnosis and pathogenesis and provides insight in activity and chronicity [2, 4], thereby influencing therapeutic decision-making and determining prognosis.

Almost all renal diseases are orphan diseases. The small number of cases is an obstacle to gather experience for nephrologists and pathologists, facing the overlap and variety of clinical presentations, the complexity of histologic patterns and the many additional clinical data and laboratory values that are needed to interpret kidney biopsies adequately. The rarity of renal diseases also hinders the collection of a sufficient number of cases for research [3]. This is why nephrology and renal pathology often are practiced in larger hospitals or hospital networks with regional or national collaborations. Even if networks and collaborations greatly facilitate teaching, research and policy-making, there is still need for large patient and biopsy series in order to better understand kidney disease and optimize treatment and care. As a result, kidney biopsy registries have been established.

Registries compile knowledge, foster collaboration and provide research data. Medical registries systematically collect a defined set of data from patients with specific health characteristics in a central database for a specific purpose [5]. Several clinical kidney registries exist, of which the United States Renal Data System and the ERA-EDTA Registry are probably the best known [6, 7]. However, these registries focus on chronic kidney disease and renal replacement therapy and mainly collect clinical diagnoses and clinical data. The scope of these registries is not kidney biopsy diagnosis or pathology data. In comparison to these well-known big clinical registries, little is known about the number, the size and the geographic distribution of specific kidney biopsy registries.

If little data exist about kidney biopsy registries, the coding practice of these registries is even less known. A coding system eliminates the variability inherent to spoken or written language and thus can be used to store, aggregate and exchange data. In the context of kidney biopsy registries, important information from a pathology report will be stored as codes. Primarily this applies to the pathology diagnosis, but also morphologic findings or reaction patterns might be coded. If the joint usage of a coding system is crucial for aggregation and exchange of data on rare diseases, it seems strange that so little is known about coding systems used by kidney biopsy registries.

In view of these issues, our study aims to (1) give an overview over kidney biopsy registries, (2) explore how these registries code renal disease and (3) identify needs for improvement of coding practice.

## Methods

### Literature search

A PubMed search was undertaken in order to find kidney biopsy registries, with specifications ‘kidney OR renal AND registry AND biopsy’. The search was last updated on 29th March 2019.

A first selection round screened the papers on the basis of their title. Articles were excluded based on the following criteria: review articles, articles about transplantation registries, renal registries based exclusively on clinical data, oncological registries and single center registries. We also excluded local or national pathology databases recording pathology diagnoses in general, however not specifically dedicated to medical kidney disease. These databases usually lack other characteristics of kidney biopsy registries such as yearly reports, publications or a dedicated webpage to renal disease. The Danish National Pathology Data Bank (Patobank) was kept as the only such database, as the former Danish Renal Biopsy Registry was incorporated into this database and kidney biopsy data continued to be published [8]. Inactive registries or temporary registries were excluded as well. Articles from or about registries that at least spanned a defined geographical region (regional or national kidney biopsy registries) were withheld. In this first selection round, we found 2 renal registries that did not record any pathology data and 3 major research consortia that will not be further discussed here because they met the exclusion criteria.

In a second selection round, remaining articles where screened using the same criteria on the basis of abstract and full text. We did not need to apply language restriction: all titles and abstracts were available in English. The search was complemented by information from personal contacts.

The same search was also run on the Cochrane library.

### Questionnaire

An online questionnaire consisting of nine questions was developed for identifying characteristics of kidney biopsy registries and for evaluating how kidney biopsy registries code (see Supplementary material appendix 1). The questionnaire contained both multiple choice questions and open questions. Questions 1 to 7 yielded easy-to-present results, whereas questions 8 and 9 needed more qualitative interpretation. In order to better understand coding systems and lists, we gave registries an example of a diagnosis/conclusion from a pathology report (mesangioproliferative glomerulonephritis, IgA nephropathy, M1 E0 S1 T0 C1, see Supplementary material appendix 1), with the question to code this according to their current practice. In addition, in an open question format we encouraged respondents to provide us with suggestions for the future.

All kidney biopsy registries were contacted by AD or SL via an email to a contact person either found on the registry website, in published articles or by personal contacts. The contact persons were given information about the study project, and were kindly asked to answer the online questionnaire via a link provided in the email. When no reply was received, after 2 weeks a reminder email was sent. Answers to the questionnaire were analyzed by AD and SL. When present, discrepancies were discussed and resolved. As a second approach, we investigated the papers published by the registries and websites -where available- to obtain information on the registries and to understand their coding systems.

Methods were carried out in accordance with relevant guidelines and regulations. Because this study did not involve experimental protocols nor the collection, use or transmission of individually identifiable data, institutional ethics committee review or approval was not required. Similarly, patient informed consent was not applicable to this study since the collection of data via the questionnaire did not involve patient data but collection of registry data.

## Results

### Literature search

**Fig. 1:**
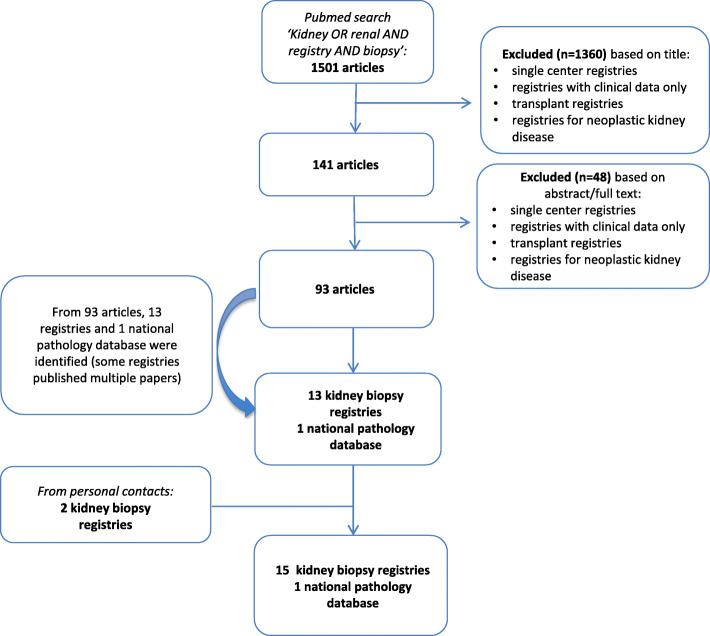
Graphical representation of the search strategy to identify medical literature about kidney biopsy registries

The literature search retrieved 1501 articles (Fig. 1). The first selection round resulted in 141 articles, the second one in 93 articles. From these we identified 14 kidney biopsy registries (13 are kidney biopsy registries proper and 1 is a national pathology database; we will for simplicity use the umbrella term ‘registries’ for all). Through personal contacts, additional 2 registries could be identified, bringing the total number of kidney biopsy registries to start with to 16. A second search on the Cochrane library retrieved 193 articles, but screening based on title did not withhold a single article.

In Table 1, we give an overview of available websites, annual reports and 1-2 relevant research papers from the identified registries, typically one paper describing the set-up of the registry and one more recent paper.

**Table 1:** Overview of websites, key publications (2 are shown) and data reports of national and/or regional kidney biopsy registries included in this study

	Identified registries or data repositories	Website	Publications (see references)	Data Reports
1	British Columbia Glomerulonephritis Network	http://www.bcrenalagency.ca/health-professionals/professional-resources/promis	[9, 10]	–
2	Czech Registry of Renal Biopsies	http://www.nefrol.cz/en/experts/renal-biopsy-registry	[11]	–
3	Flemish Collaborative Glomerulonephritis Group Registry	http://www.nbvn.be/blog/organisatie/fcgg-in-english	[12]	https://www.renalconference2018.com/images/Presentaties/Zaterdag/Wim_Laurens-Ame%CC%81lie_Dendooven%E2%80%93FCGG_Flemisch_Collaborative_Glomerulonephritis_Group_Current_status_of_the_Registry_and_Future_Perspectives.pdf
4	Italian Registry of Renal Biopsies	http://www.irrb.net/	[13, 14]	–
5	Japanese Renal Biopsy Registry (J-RBR)	–	[15, 16].	Japan Renal Biopsy Registry and Japan Kidney Disease Registry: Committee Report for 2009 and 2010. Sugiyama H et al. Clin Exp Nephrol. 2013;17:155–73.
6	Limburg Renal Registry	–	[17, 18]	–
7	Malaysian Registry of Renal Biopsy	https://www.macr.org.my/emrrb/zAu_login.jsp	–	https://www.msn.org.my/nrr/mrrb_report.jsp
8	National Pathology Database Denmark (PATOBANK)	http://www.patobank.dk/index.php?ID=1&lang=da	[19, 20]	https://www.patobank.dk/
9	Norwegian Renal Registry	http://www.nephro.no/nnr.html	[21, 22]	https://www.nephro.no/nnr/AARSRAPPORT_NNR_2018_ToC.pdf
10	Philippine Renal Disease Registry	http://www.nkti.gov.ph/index.php/services/specialty-centers/renal-disease-control-program-redcop	–	On request via National Kidney and Transplantation Initiative, Philippines
11	Polish Registry of Kidney Biopsies	–	[23, 24]	–
12	Scottish Renal Biopsy Registry	http://www.srr.scot.nhs.uk/Biopsy-Registry/Main.html	[25, 26]	https://www.srr.scot.nhs.uk/Biopsy-Registry/Main.html
13	Spanish Renal Registry	https://www.senefro.org/modules.php?name=home&lang=ES	[27, 28]	https://www.senefro.org/contents/webstructure/REGN2019_2_.pdf
14	Swedish Renal Registry	https://www.medscinet.net/snr/default.aspx?lang=1	[29, 30]	https://www.medscinet.net/snr/rapporterdocs/Svenskt%20Njurregister%20A%CC%8Arsrapport%202019.pdf
15	Uruguayan Registry of Glomerular Diseases	–	[31, 32]	https://www.nefrologia.hc.edu.uy/index.php/prevencion-glomerulopatias
16	National Renal Biopsy Registry, Taiwan	https://www.tsn.org.tw/enVersion/about.aspx	[33]	–

**Fig. 2:**
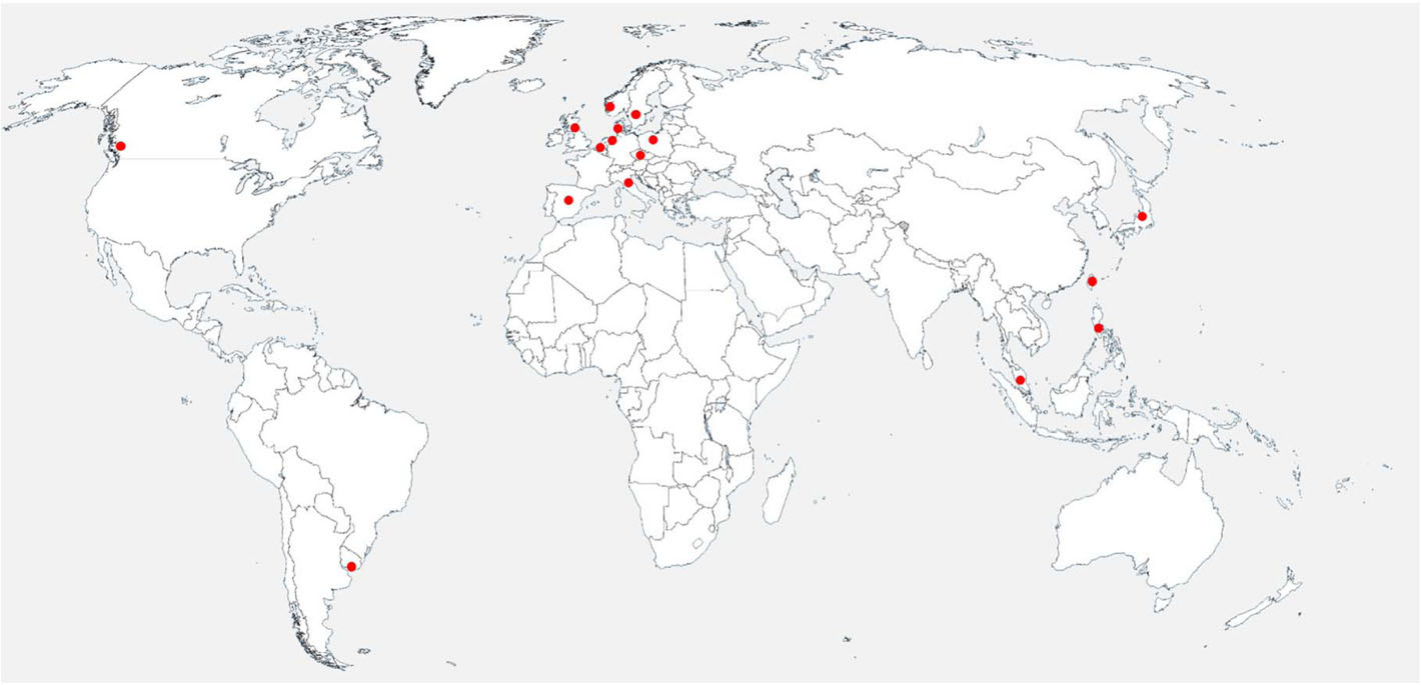
Geographical distribution of kidney biopsy registries around the globe (the basis map was reused from publicdomainvectors.org)

Most of the registries were located in Europe (*n* = 10; Fig. 2). There was 1 registry in North America (Canada), 1 in South America (Uruguay) and 4 in Asia (Japan, Malaysia, The Philippines, Taiwan).

### Questionnaire

Fifteen of 16 registries filled out the online questionnaire, thus the response rate was 94%. Three of these 15 responding registries proved to be regional renal biopsy registries, while 12/15 were organized in a national fashion. Data for the one registry that did not respond were analyzed based on published literature [33].

#### What type of information do registries collect?

All registries collect the pathology diagnosis, as this was an inclusion criterion for this study. Fourteen of 16 collect the clinical diagnosis for the same biopsy episode. Fifteen of 16 registries record clinical data related to the kidney biopsy diagnosis (such as blood pressure) and 11/16 pathology data related to the kidney biopsy diagnosis (such as number of glomeruli). Eight of 16 registries collect clinical data related to the transplant diagnosis (such as post-transplant serum creatinine levels) and 3/16 registries collect pathology data related to transplant biopsies (such as degree of interstitial fibrosis) (Table 2).

**Table 2:** Overview over kidney biopsy registries. Data are based on an online questionnaire except for one registry

	Registry	Data collection	Diagnosis registered	Tx included	Coverage	In use (years)	Coding system					Who codes	Self-reported satisfaction (0–5)
							Proprietary	ERA-EDTA	SNO-MEDSNOMED (older version)	SNOMED CT	ICD-9		
1	British Columbia Glomerulonephritis Registry	C, P	C, P	No	Regional	5–10	x					Nephrologist	4
2	Czech Registry of Renal Biopsies	C	C, P	No	National	> 20	x					Nephrologist	4
3	Flemish Collaborative Glomerulonephritis Registry	C, P	C, P	No	Regional	< 5	x	x				Nephrologist Pathologist	4
4	Italian Registry of Renal Biopsies	C, P	C, P	Yes^a^	National	> 20		x				Nephrologist Pathologist	3
5	Japanese Renal Biopsy Registry	C	C, P	Yes^a^	National	11–20	x					No data	3
6	Limburg Renal Registry (Netherlands)	C, P	C, P	Yes^a^	Regional	> 20	x		x		x	Pathologist	3
7	Malaysian Registry of Renal Biopsies	C, P	C, P	Yes^a^	National	11–20	x					Nephrologist	4
8	National Pathology Databank Denmark (PATOBANK)	No data	P	Yes	National	11–20			x^c^	x		Pathologist	2
9	Norwegian Renal Registry	C, P	C, P	No	National	> 20	x	x				Nephrologist Pathologist	3
10	Philippine Registry of Glomerular Disease	C, P	C, P	Yes	National	11–20	x					Nephrologist Pathologist	4
11	Polish Registry of Renal Biopsies	C	P	No	National	5–10	x					Pathologist	No data
12	Scottish Renal Biopsy Registry	C, P	C, P	Yes	National	11–20		x^b^				Nephrologist	3
13	Spanish Registry of Glomerulonephritis	C, P	C, P	No	National	> 20	x					Nephrologist	4
14	Swedish Renal Registry	C, P	C, P	No	National	< 5		x	x			Nephrologist	4
15	Uruguayan Registry of Glomerular diseases	C, P	C, P	No	National	> 20	x					Nephrologist Pathologist	4
16	National Renal Biopsy Registry Taiwan^d^	C	C,P	Yes^a^	National	5–10	x					Nephrologist	No data

C clinical data, P pathology data. ^a^Only clinical data, no pathological data related to Tx; ^b^Proprietary codes based on ERA-EDTA PRD; ^c^SNOMED (older version), but mapped to SNOMED CT; ^d^Did not participate in the online questionnaire; data based on published paper [33]

#### How much experience do registries have?

There is a wide variability in the age of these registries. However, most registries exist for a long time and thus have a strong experience base: 11/16 exist for more than 10 years and 6/16 are even more than 20 years old. Two registries have recently been established (< 5 years).

#### How do registries code kidney biopsy diagnoses?

When it comes to coding practice, a common line is that either nephrologist (7/16) or pathologist (3/16) or both (5/16) code. Two registries have additional coding assistance, in one by an administrator/study nurse, in another by an informatician or coding expert and an epidemiologist. (Table 2).

Looking at coding systems, most registries use a proprietary coding list to register diagnoses, either exclusively (9/16) or in combination with another coding system (3/16). One registry uses SNOMED (both an older SNOMED version and SNOMED CT). Two registries use the ERA-EDTA PRD [34]. Two more registries use a combination of a proprietary coding list and the ERA-EDTA PRD system. One registry uses a proprietary list, ICD-10, SNOMED (older version) and SNOMED CT.

In general, registries are relatively satisfied with the (often proprietary) system they use, with 9 of 14 reporting a 4 for user satisfaction on a scale of 0 to 5, where 0 means ‘totally unsatisfied’ and 5 means ‘very satisfied’.

Next, registries were asked to code a typical diagnosis of IgA nephropathy M1 E0 S1 T0 C1. The responses illustrate the diversity of systems in use (Table 3). Often (combinations of) letters and ciphers serve as codes for predefined diagnostic terms, as in the ERA-EDTA PRD codes used by 5/15 registries and in some proprietary coding list (e.g. British Columbia, Poland and Flanders). In fact, the largest number of registries using the same code value is 5, which are the registries using the ERA EDTA PRD codes. Of course, code values from registries with proprietary coding systems are different. However, one could assume that code terms for the concept ‘IgA nephropathy’ are at least the same. This is not the case; even if all registries code for the diagnosis ‘IgA nephropathy’, only 5/14 registries with proprietary coding systems use the exact identical code term ‘IgA nephropathy’. Examples for variations and specifications used are ‘IgA nephritis’, ‘IgA nephropathy, primary’ and ‘IgA nephropathy with crescents’.

**Table 3:** Coding practice in kidney biopsy registries in this study

	Registry	Coding system	Code(s)	Code text
1	British Columbia Glomerulonephritis Network	Proprietary	G23.1 V3	IgA nephropathy-primary Hypertensive/benign/ischemic nephrosclerosis
2	Czech Registry of Renal Biopsies	Proprietary	1730	IgA nephropathy with crescents
3	Flemish Collaborative Glomerulonephritis Group Registry	Proprietary (FCGG-NBVN) ERA-EDTA PRD	3a 1128	IgA nephropathy, primary IgA nephropathy-histologically proven
4	Italian Registry of Renal Biopsies	ERA-EDTA PRD	1128	IgA nephropathy-histologically proven
5	Japanese Renal Biopsy Registry (J-RBR)	Proprietary	–	IgA nephropathy (histological diagnosis by pathogenesis) Mesangial proliferative glomerulonephritis (histological diagnosis by histopathology)
6	Limburg Renal Registry	Proprietary	–	Mesangioproliferative glomerulonephritis IgA nephropathy Interstitial fibrosis Arteriosclerosis
7	Malaysian Registry of Renal Biopsy	Proprietary	–	IgA nephropathy
8	National Pathology Database Denmark (PATOBANK)	SNOMED (older version)	T71000 M46862 S67300 M53300 M58000	Kidney Diffuse mesangial proliferation IgA nephritis Glomerulosclerosis Tubular atrophy
9	Norwegian Renal Registry	ERA-EDTA PRD NNR 2013 NNR 2011	1128 300 3	IgA nephropathy-histologically proven IgA nefropati IgA nefropati
10	Philippine Renal Disease Registry	Proprietary	–	Mesangioproliferative glomerulonephritis IgA nephropathy
11	Polish Registry of Kidney Biopsies	Proprietary	124	Class IV (diffuse proliferative) lesions according to Haas classification in IgA Nephropathy
12	Scottish Renal Biopsy Registry	ERA-EDTA PRD	1128	IgA nephropathy-histologically proven
13	Spanish Renal Registry	Proprietary	–	IgA nephropathy
14	Swedish Renal Registry	ERA-EDTA PRD SNOMED (older version)	1128 M46862 D45870 M5440 M52200 M52220	IgA nephropathy-histologically proven
15	Uruguayan Registry of Glomerular Diseases	Proprietary	1151	IgA nephropathy

We asked contact persons to code the following situation:

Biopsy: 15 glomeruli, 1 cellular crescent, 2 lesions of segmental glomerulosclerosis, 4 lesions of global glomerulosclerosis. Tubular atrophy in around 20% of the cortical area. Moderate arteriolosclerosis and arteriolosclerosis. IH: dominant IgA positivity. Diagnosis: mesangioproliferative glomerulonephritis; IgA nephropathy; Oxford classificaion: M1 E0 S1 T0 C1

Data are depicted literally as mentioned by the registry contact persons

The responses also illustrate various coding policies. Looking at the proprietary coding systems, in some instances only the diagnosis itself is coded (e.g. Malaysia and Spain), in other instances also morphology data are rendered in a code (e.g. Czech Republic, Japan, Limburg, Philippines and Poland). The same holds true for the established coding systems: in ERA-EDTA PRD the diagnosis as such is coded (‘IgA nephropathy’), whereas in the (older versions of) SNOMED, histologic patterns (‘Diffuse mesangial proliferation’ or ‘Tubular atrophy’) are coded as well.

#### Self-reported problems and suggestions for improvement

In an open question format, we asked the contact persons from the registries about the advantages and disadvantages of their system. Eleven of 15 registries answered this question with a comment on problems of their registry or with a suggestion for improvement.

The lack of maintenance and updates of the coding system was the most common comment, mentioned in some form by respondents from 6 registries. Two registries mentioned a problem with interoperability with international systems. Other issues were mentioned once: ambiguity related to lack of coding rules, insufficient coding possibilities (not all diagnoses included, no morphology diagnoses, too many irrelevant codes), difficulties retrieving data from the system, problems to code more than one diagnosis per biopsy, necessity of a system for transplant biopsies and finally the need to record histopathological patterns and findings.

Suggestions for improvement included the need for a simple system (mentioned twice), the need for a consistent system (mentioned once) and finally the need to code prospectively (mentioned once): this means coding when making the diagnosis and not when data are recorded in the registry (i.e. retrospectively).

## Discussion

In this paper, we give an overview over national and regional kidney biopsy registries and combine a literature search with an online questionnaire to research registries’ current way of kidney biopsy coding. Additionally, we report suggestions for improvement of coding practice.

First of all, we note that most kidney biopsy registries are situated in European countries and some in Asian countries (Fig. 2). There is one more registry in North America (Canada) and one in South America (Uruguay). In contrast to the high number of registries in Europe, there are large geographical areas without a single kidney biopsy registry. This is comprehensible in areas like sub-Saharan Africa, where resources for the collection and processing of kidney biopsies might be limited [35]. On the other hand, it is surprising that highly populated countries and/or countries with a high standard of healthcare systems such as the USA, China or India do not have established kidney biopsy registries covering a defined region on the national or regional level [36, 37]. A substitute might be large-scale single center registries, which often serve as tertiary referral centers. Prominent examples are the Toronto Glomerulonephritis Registry, the Renal Biopsy Laboratory (Department of Laboratory Medicine and Pathology) at Mayo Clinic or the Division of Nephropathology at the University of North Carolina at Chapel Hill [38–40]. A second alternative might be large research consortia (for example the NEPTUNE in the USA, just to mention one) that might fill in this gap [41]. However, research consortia are often more focused on thematic research than epidemiologic research. Finally, another possible substitute are end-stage renal disease registries [42]. For example, the US Renal Data System (USRDS) registers diagnoses of patients with end-stage renal disease using ICD-9 and ICD-10 codes [6]. However, since end-stage renal disease registries do not incorporate pathology diagnoses, the registry data do not necessarily reflect the kidney biopsy diagnoses, and certainly not important morphological patterns or other key changes. Another drawback is that only patients with ESRD are covered. Since many renal diseases do not lead to end-stage renal failure, data about these diseases are thus lost. For a review on renal registries proper, we refer to the paper of Liu et al. [42].

As an alternative to registries, epidemiologic data related to kidney biopsies might be collected retrospectively [36]. There are plenty of such publications. However, these publications usually present epidemiologic data on a single-center, regional or national basis at a certain time point or over a restricted time period. There is no prospective data collection or continuous monitoring of biopsy data as it would be provided by a registry. Therefore, even if these retrospective analyses add valuable knowledge, they are not really an alternative for proper kidney biopsy registries which can follow epidemiological developments over a long period of time using a consistent dataset [43].

Not only registry number in a defined geographical area is variable, there is also a variety in set-up of the registries, which sometimes include transplant biopsies, but more often do not. In many registries, nephrologists and pathologists collaborate and it varies from registry to registry if the nephrologist codes or the pathologist codes or both.

It is noteworthy how old many registries are. These old registries allow for studies about the long-term course of kidney disease and underline the usefulness of kidney biopsy registries [44, 45]. The age of the registries is also reflected by the coding systems and mappings used. This is exemplified by the use of or mapping to older and no longer updated SNOMED versions in Denmark, Sweden or the Netherlands. In fact, lack of maintenance and updates of coding systems was the most common problem reported by the registries.

Registries where the nephrologists play a central role, especially in Europe, often map to ERA-EDTA PRD. This is understandable, as ERA-EDTA PRD is the uniform coding system for dialysis registries in Europe [34] and thus, is a known tool for registering renal disease. However, using ERA-EDTA PRD for biopsies can be challenging, as many morphology-based diagnoses are lacking in ERA-EDRA PRD (this was also one of the comments brought forward by the registries).

Most registries use proprietary coding systems. In this study, we did not investigate specifically why people prefer proprietary coding systems. It is very likely, though, that the use of proprietary coding systems is related to the fact that many international terminologies are not designed for pathology purposes. International coding systems covering pathology needs such as SNOMED and SNOMED CT are highly complex. Practical application of these systems might be hampered due to heterogeneity or lack of renal disease classifications in the past. Another reason could be the way registries are established and managed. This is often done by enthusiastic committed medical professionals [19]. Informaticians and coding experts are probably rarely involved. At least, our survey shows in terms of coding, that only one registry had coding assistance by an informatician or coding expert and an epidemiologist.

Sometimes these proprietary systems are mapped to international terminologies, though often they are not. Clearly, data collection in regional or national registries in itself is a tool to standardize coding of diagnoses from different centers. However, when no mapping is available, the downsides of proprietary coding systems are obvious: since clinical concepts have many synonyms, the terms chosen by registries for a specific concept will be variable. Of course, code values will differ from system to system. Therefore, there is no means to easily exchange data with other registries, countries or even consortia.

Our coding task for registries “kidney biopsy with IgA nephropathy” highlights these difficulties. As IgA nephropathy is one of the most common nephropathies worldwide [46], the entity is relatively straightforward to code and this coding task is a daily routine in all registries. However, comparison or aggregation based on key information – the diagnosis IgA nephropathy - is not possible without profound manual interaction because terms used are different and a unique code is missing. If already a common entity like IgA nephropathy requires manual interaction to aggregate data, it is easy to anticipate how difficult it would be to compare and aggregate data from rare kidney diseases, morphological patterns or key histological findings. These observations clearly underline the necessity for a common coding system.

In addition, the coding task reveals a second challenge. Some registries only code the main diagnosis “IgA nephropathy” while others code additional morphological findings. For example, a minority of registries codes the morphological reaction pattern. Moreover, if registries code for morphological patterns, then they do it in different ways. The example illustrates that an investigation of data from several registries concerning morphological reaction patterns in a particular disease would not yield reliable data. Consequently, to ensure the best use of registry data, it would be advisable to establish coding rules in addition to a common coding system.

As renal pathologists use morphological patterns as a basis of diagnostic categorization, apart from clinical correlations, it is not difficult to conceive that systems originally designed for mortality statistics (such as ICD) or for registering end-stage renal disease (such as ERA-EDTA PRD) are imperfect for registering biopsy diagnoses. Thus, this research emphasizes the need for a consensus coding system that can be used by pathologists and that maps to other, more general and interchangeable, health terminology systems.

The present study has several limitations. First, the study is constrained to regional or national kidney biopsy registries. We excluded the investigation of research consortia or time-limited research registries, single center registries and pathology databases. Second, kidney biopsy registries that do not actively publish or maintain a website may have remained undetected. However, it is unlikely that such registries would have adopted any well-established renal pathology coding system.

In conclusion, our study shows large gaps in kidney biopsy registry coverage around the globe. Among existing kidney biopsy registries, there is limited use of international coding systems, hampering comparison of findings and aggregation of data. One reason might be the perceived lack of a coding system suitable for kidney biopsies. Another main reason is the long lifespan of many kidney biopsy registries, which makes continuous updating of coding systems in relation to knowledge increase challenging. There is a need for an international coding system that meets the needs of kidney biopsy registries in order to utilize the potential of these registries.

## Supplementary Information

**Additional file 1 Appendix 1.** Online questionnaire sent to kidney biopsy registries.

## Data Availability

Most data are represented in the paper; complete data from the current study are however available from the corresponding author on reasonable request.

## References

[1] Radhakrishnan J, Cattran DC (2012). The KDIGO practice guideline on glomerulonephritis: reading between the (guide)lines--application to the individual patient. Kidney Int.

[2] Srivastava A, Palsson R, Kaze AD, Chen ME, Palacios P, Sabbisetti V, et al. The prognostic value of histopathologic lesions in native kidney biopsy specimens: results from the Boston kidney biopsy cohort study. J Am Soc Nephrol. 2018;29(8):2213–24. 10.1681/ASN.2017121260. 10.1681/ASN.2017121260 PMC6065095 29866798

[3] Leaf DE, Appel GB, Radhakrishnan J (2010). Glomerular disease: why is there a dearth of high quality clinical trials?. Kidney Int.

[4] Sethi S, D'Agati VD, Nast CC, Fogo AB, De Vriese AS, Markowitz GS (2017). A proposal for standardized grading of chronic changes in native kidney biopsy specimens. Kidney Int.

[5] Arts DG, De Keizer NF, Scheffer GJ (2002). Defining and improving data quality in medical registries: a literature review, case study, and generic framework. J Am Med Inform Assoc.

[6] Saran R, Robinson B, Abbott KC, Bragg-Gresham J, Chen X, Gipson D (2020). US Renal Data System 2019 Annual Data Report: Epidemiology of Kidney Disease in the United States. Am J Kidney Dis.

[7] Kramer A, Pippias M, Noordzij M, Stel VS, Andrusev AM, Aparicio-Madre MI, Arribas Monzón FE, Åsberg A, Barbullushi M, Beltrán P, Bonthuis M, Caskey FJ, Castro de la Nuez P, Cernevskis H, de Meester J, Finne P, Golan E, Heaf JG, Hemmelder MH, Ioannou K, Kantaria N, Komissarov K, Korejwo G, Kramar R, Lassalle M, Lopot F, Macário F, Mackinnon B, Pálsson R, Pechter Ü, Piñera VC, Santiuste de Pablos C, Segarra-Medrano A, Seyahi N, Slon Roblero MF, Stojceva-Taneva O, Vazelov E, Winzeler R, Ziginskiene E, Massy Z, Jager KJ (2019). The European renal association - European Dialysis and transplant association (ERA-EDTA) registry annual report 2016: a summary. Clin Kidney J.

[8] Heaf JG, Hansen A, Laier GH (2018). Quantification of cancer risk in glomerulonephritis. BMC Nephrol.

[9] Canney M, Induruwage D, Sahota A, McCrory C, Hladunewich MA, Gill J, Barbour SJ (2020). Socioeconomic position and incidence of glomerular diseases. Clin J Am Soc Nephrol.

[10] Barbour S, Beaulieu M, Gill J, Djurdjev O, Reich H, Levin A (2013). An overview of the British Columbia glomerulonephritis network and registry: integrating knowledge generation and translation within a single framework. BMC Nephrol.

[11] Maixnerova D, Jancova E, Skibova J, Rysava R, Rychlik I, Viklicky O, Merta M, Kolsky A, Reiterova J, Neprasova M, Kidorova J, Honsova E, Tesar V (2015). Nationwide biopsy survey of renal diseases in the Czech Republic during the years 1994-2011. J Nephrol.

[12] Dendooven A, Helbert M, De Paepe P, Lerut E, De Vriese AS, Nguyen TQ. Development of a new renal pathology coding list for the Flemish Renal Biopsy (FCGG) Registry. Nephrology Dialysis Transplantation. 2017;32:457.

[13] Gesualdo L, Di Palma AM, Morrone LF, Strippoli GF, Schena FP (2004). The Italian experience of the national registry of renal biopsies. Kidney Int.

[14] Schena FP (1997). Survey of the Italian registry of renal biopsies. Frequency of the renal diseases for 7 consecutive years. The Italian Group of Renal Immunopathology. Nephrol Dial Transplant.

[15] Sugiyama H, Yokoyama H, Sato H, Saito T, Kohda Y, Nishi S (2011). Japan renal biopsy registry: the first nationwide, web-based, and prospective registry system of renal biopsies in Japan. Clin Exp Nephrol.

[16] Okabayashi Y, Tsuboi N, Amano H, Miyazaki Y, Kawamura T, Ogura M, Narita I, Ninomiya T, Yokoyama H, Yokoo T (2018). Distribution of nephrologists and regional variation in the clinical severity of IgA nephropathy at biopsy diagnosis in Japan: a cross-sectional study. BMJ Open.

[17] Van Paassen P, Vriesman PJVB, Van Rie H, Tervaert JWC (2004). Signs and symptoms of thin basement membrane nephropathy: a prospective regional study on primary glomerular disease—the Limburg renal registry. Kidney Int.

[18] Timmermans S, Abdul-Hamid MA, Potjewijd J, Theunissen R, Damoiseaux J, Reutelingsperger CP (2018). C5b9 formation on endothelial cells reflects complement defects among patients with renal thrombotic Microangiopathy and severe hypertension. J Am Soc Nephrol.

[19] Heaf J (2004). The Danish renal biopsy register. Kidney Int.

[20] Heaf JG, Hansen A, Laier GH (2018). Quantification of cancer risk in glomerulonephritis. BMC Nephrol.

[21] Aasarod K, Iversen BM, Hammerstrom J, Bostad L, Vatten L, Jorstad S (2000). Wegener's granulomatosis: clinical course in 108 patients with renal involvement. Nephrol Dial Transplant.

[22] Tondel C, Vikse BE, Bostad L, Svarstad E (2012). Safety and complications of percutaneous kidney biopsies in 715 children and 8573 adults in Norway 1988-2010. Clin J Am Soc Nephrol.

[23] Perkowska-Ptasinska A, Deborska-Materkowska D, Bartczak A, Stompor T, Liberek T, Bullo-Piontecka B, Wasinska A, Serwacka A, Klinger M, Chyl J, Kuriga M, Malecki R, Marczewski K, Hryniewicz B, Gregorczyk T, Wieliczko M, Niemczyk S, Rostkowska O, Paczek L, Durlik M (2016). Kidney disease in the elderly: biopsy based data from 14 renal centers in Poland. BMC Nephrol.

[24] Perkowska-Ptasinska A, Bartczak A, Wagrowska-Danilewicz M, Halon A, Okon K, Wozniak A (2017). Clinicopathologic correlations of renal pathology in the adult population of Poland. Nephrol Dial Transplant.

[25] McQuarrie EP, Mackinnon B, Young B, Yeoman L, Stewart G, Fleming S (2009). Centre variation in incidence, indication and diagnosis of adult native renal biopsy in Scotland. Nephrol Dial Transplant.

[26] McQuarrie EP, Mackinnon B, McNeice V, Fox JG, Geddes CC (2014). The incidence of biopsy-proven IgA nephropathy is associated with multiple socioeconomic deprivation. Kidney Int.

[27] Gutierrez E, Praga M, Rivera F, Sevillano A, Yuste C, Goicoechea M, et al. Changes in the clinical presentation of immunoglobulin A nephropathy: data from the Spanish registry of glomerulonephritis. Nephrol Dial Transplant. 2018;33(3):472–7. 10.1093/ndt/gfx058. 10.1093/ndt/gfx058 28460086

[28] Rivera F, Lopez-Gomez JM, Perez-Garcia R. Spanish registry of G. frequency of renal pathology in Spain 1994-1999. Nephrol Dial Transplant. 2002;17(9):1594–602. 10.1093/ndt/17.9.1594. 10.1093/ndt/17.9.1594 12198210

[29] Weiner M, Bjorneklett R, Hruskova Z, Mackinnon B, Poulton CJ, Sindelar L (2019). Proteinase-3 and myeloperoxidase serotype in relation to demographic factors and geographic distribution in anti-neutrophil cytoplasmic antibody-associated glomerulonephritis. Nephrol Dial Transplant.

[30] Peters B, Nasic S, Segelmark M. Clinical parameters predicting complications in native kidney biopsies. Clin Kidney J. 2019;13(4):654–9. 10.1093/ckj/sfz132. 10.1093/ckj/sfz132 PMC7467621 32905412

[31] Garau M, Cabrera J, Ottati G, Caorsi H, Gonzalez Martinez F, Acosta N, Aunchayna MH, Gadola L, Noboa O (2018). Temporal trends in biopsy proven glomerular disease in Uruguay, 1990-2014. Plos One.

[32] Mazzuchi N, Acosta N, Caorsi H, Schwedt E, Di Martino LA, Mautone M (2005). Frequency of diagnosis and clinic presentation of glomerulopathies in Uruguay. Nefrologia..

[33] Chiu HF, Chen HC, Lu KC, Shu KH (2018). Taiwan Society of N. Distribution of glomerular diseases in Taiwan: preliminary report of National Renal Biopsy Registry-publication on behalf of Taiwan Society of Nephrology. BMC Nephrol.

[34] Venkat-Raman G, Tomson CR, Gao Y, Cornet R, Stengel B, Gronhagen-Riska C, Reid C, Jacquelinet C, Schaeffner E, Boeschoten E, Casino F, Collart F, de Meester J, Zurriaga O, Kramar R, Jager KJ, Simpson K, ERA-EDTA Registry (2012). New primary renal diagnosis codes for the ERA-EDTA. Nephrol Dial Transplant.

[35] Okpechi IG, Ameh OI, Bello AK, Ronco P, Swanepoel CR, Kengne AP (2016). Epidemiology of histologically proven glomerulonephritis in Africa: a systematic review and meta-analysis. Plos One.

[36] Al Turk AA, Estiverne C, Agrawal PR, Michaud JM (2018). Trends and outcomes of the use of percutaneous native kidney biopsy in the United States: 5-year data analysis of the Nationwide inpatient sample. Clin Kidney J.

[37] Hou JH, Zhu HX, Zhou ML, Le WB, Zeng CH, Liang SS (2018). Changes in the Spectrum of kidney diseases: an analysis of 40,759 biopsy-proven cases from 2003 to 2014 in China. Kidney Dis (Basel).

[38] Cattran DC, Rao P (1998). Long-term outcome in children and adults with classic focal segmental glomerulosclerosis. Am J Kidney Dis.

[39] O'Shaughnessy MM, Hogan SL, Poulton CJ, Falk RJ, Singh HK, Nickeleit V (2017). Temporal and demographic trends in glomerular disease epidemiology in the southeastern United States, 1986-2015. Clin J Am Soc Nephrol.

[40] Ravindran A, Fervenza FC, Smith RJH, De Vriese AS, Sethi S (2018). C3 Glomerulopathy: ten Years’ experience at Mayo Clinic. Mayo Clin Proc.

[41] Gadegbeku CA, Gipson DS, Holzman LB, Ojo AO, Song PX, Barisoni L (2013). Design of the Nephrotic Syndrome Study Network (NEPTUNE) to evaluate primary glomerular nephropathy by a multidisciplinary approach. Kidney Int.

[42] Liu FX, Rutherford P, Smoyer-Tomic K, Prichard S, Laplante S (2015). A global overview of renal registries: a systematic review. BMC Nephrol.

[43] Pesce F, Schena FP (2010). Worldwide distribution of glomerular diseases: the role of renal biopsy registries. Nephrol Dial Transplant.

[44] Knoop T, Vikse BE, Mwakimonga A, Leh S, Bjorneklett R (2017). Long-term outcome in 145 patients with assumed benign immunoglobulin a nephropathy. Nephrol Dial Transplant.

[45] Kunter U, Floege J (2017). The longer the better: follow-up in seemingly ‘benign’ immunoglobulin a nephropathy. Nephrol Dial Transplant.

[46] O'Shaughnessy MM, Hogan SL, Thompson BD, Coppo R, Fogo AB, Jennette JC. Glomerular disease frequencies by race, sex and region: results from the International Kidney Biopsy Survey. Nephrol Dial Transplant. 2018;33(4):661–9. 10.1093/ndt/gfx189. 10.1093/ndt/gfx189 PMC6659026 29106637

