# Supplementary material for: Coding practice in national and regional kidney biopsy registries
Source: BMC Nephrol. 2021 May 24;22:193. doi: 10.1186/s12882-021-02365-3 (PMC8146626; doi:10.1186/s12882-021-02365-3)
Supplement: Supplementary file 1 — Additional file 1 Appendix 1. Online questionnaire sent to kidney biopsy registries. [file 12882_2021_2365_MOESM1_ESM.docx]

# Supplementary material

**Coding practice in national and regional kidney biopsy registries**

Amélie Dendooven^1^, Han Peetermans^2^, Mark Helbert^2^, Tri Q. Nguyen^3^, Niels Marcussen^4^, Michio Nagata^5^, Loreto Gesualdo^6^, Agnieszka Perkowska-Ptasinska^7^, Cristina Capusa ^8^, Juan M. Lopez-Gomez^9^, Colin Geddes^10^, Myrurgia Abdul-Hamid^11^, Mårten Segelmark^12^, Rosnawati Yahya^13^, Mariela Garau^14^, Russell Villanueva^15^, Anthony (Tony) Dorman^16^, Sean Barbour^17^, Ronald Cornet^18^, Helmut Hopfer^19^, Kerstin Amann^20^, Sabine Leh^21^.

On behalf of the Kidney Biopsy Codes for Pathologists project ([www.kibico.org](http://www.kibico.org))

^1^Ghent University, Belgium

^2^ZNA Middelheim Hospital, Belgium

^3^University Medical Center, Utrecht, The Netherlands

^4^Odense University Hospital, Denmark

^5^University of Tsukuba Hospital, Japan

^6^University of Bari Aldo Moro, Italy

^7^Medical University of Warsaw, Poland

^8^Carol Davila University of Medicine and Pharmacy, Romania

^9^Hospital General Universitario Gregorio Marañón, Spain

^10^Glasgow Renal and Transplant Unit, United Kingdom

^11^Maastricht University Medical Center, The Netherlands

^12^Lund University, Sweden

^13^Hospital Kuala Lumpur, Malaysia

^14^University of the Republic, Uruguay

^15^National Kidney and Transplant Institute, The Philippines

^16^Beaumont Hospital, Ireland

^17^University of British Columbia, Canada

^18^Amsterdam University Medical Center, The Netherlands

^19^University Hospital of Basel, Switzerland

^20^Universitätsklinikum Erlangen, Germany

^21^Haukeland University Hospital, Bergen, Norway

**Appendix 1: Online questionnaire sent to kidney biopsy registries**

Q1 Which data does your registry collect (multiple answers possible)?

-Clinical data related to medical kidney biopsies (e.g. blood pressure)

-Pathology data related to medical kidney biopsies (e.g. number of glomeruli)

-Clinical data related to transplant kidney biopsies (e.g. serum creatinine)

-Pathology data related to transplant kidney biopsies (e.g. score of interstitial fibrosis)

-Pathology diagnosis

-Clinical diagnosis (based on pathology diagnosis of the kidney biopsy)

-Other (please specify)

Q2 Is your registry

-nationwide

-single-center

-multicenter

-international

-other (please specify)

Q3 How long is your registry in use?

-<5 years

-5-10 years

-11-20 years

->20 years

Q4 How is the kidney biopsy diagnosis recorded in the registry?

-Free text field

-Published diagnosis list/coding system; please specify the reference of the publication

-Proprietary diagnosis list/coding system; please specify

-Other, please specify

Q5 Is your coding system compatible with or mapped to (multiple answers possible)

-SNOMED CT

-ERA-EDTA PRD

-ICD-10

-Other (please specify)

Q6 Who performs the coding for your registry? In other words, who decides which code is to be used?

-Nephrologist

-Pathologist

-Administrator/study nurse

-Informatician /coding expert

Q7 How would you rate your satisfaction with your registry’s approach of coding biopsy diagnosis as it is now, on a scale of 0 to 5, where 0 is ‘totally unsatisfied’ and 5 is ‘very satisfied’?

1 2 3 4 5

Comment: what are the advantages and disadvantages of your system? Think of ease-of-use, interoperability with other systems, user satisfaction, possibility to update…

Q8 To understand your coding system better, we would ask you to give us an example for how you code. Could you code the renal biopsy described below?
Biopsy:
15 glomeruli, 1 cellular crescent, 2 segmental glomerulosclerosis, 4 global glomerulosclerosis. Tubular atrophy in around 20% of the cortical area. Moderate arteriolosclerosis and arteriosclerosis.
IH: Dominant IgA positivity.
EM: Mesangial electron dense deposits.
Diagnosis: mesangioproliferative glomerulonephritis; IgA nephropathy.
Oxford classification: M1 E0 S1 T0 C1

Q9 What should we bear in mind with the Kidney Biopsy Codes project? Do you have suggestions for us?

…
